# miR-4443 promotes radiation resistance of esophageal squamous cell carcinoma via targeting PTPRJ

**DOI:** 10.1186/s12967-022-03818-5

**Published:** 2022-12-28

**Authors:** Xiaobo Shi, Xiaoxiao Liu, Shan Huang, Yu Hao, Shupei Pan, Yue Ke, Wei Guo, Yuchen Wang, Hongbing Ma

**Affiliations:** Department of Radiation Oncology, The Second Affiliated Hospital of Xi’an Jiaotong University, No. 157, Xi Wu Road, Xi’an, 710004 China

**Keywords:** Esophageal squamous cell carcinoma, Radioresistance, WGCNA, miR-4443/PTPRJ axis, Apoptosis

## Abstract

**Background:**

Radiotherapy is one of the main treatments for esophageal squamous cell carcinoma (ESCC), but its efficacy is limited by radioresistance. MicroRNAs play a crucial role in posttranscriptional regulation, which is linked to the cancer response to radiation.

**Methods:**

We successfully established a radioresistant cell line model by using fractionated irradiation. qRT-PCR was adopted to detect the expression of miR-4443 in human normal esophageal cell lines, tumor cells, and radioresistant cells. Next, CCK-8, colony formation, apoptosis, and cell cycle assays were used to assess the biological effect of miR-4443. Weighted gene coexpression network analysis (WGCNA) was performed to identify potential radiosensitivity-related genes. Additionally, we predicted the probable targets of the miRNA using bioinformatic methods and confirmed them using Western blot.

**Results:**

miR-4443 was significantly upregulated in radioresistant ESCC cells. Enhancement of miR-4443 further decreased the radiosensitivity of ESCC cells, while inhibition of miR-4443 increased the radiosensitivity of ESCC cells. Notably, miR-4443 modulated radiosensitivity by influencing DNA damage repair, apoptosis, and G2 cycle arrest. By using WGCNA and experimental validation, we identified PTPRJ as a key target for miRNA-4443 to regulate radiosensitivity. The effects of miR-4443 overexpression or inhibition could be reversed by increasing or decreasing PTPRJ expression.

**Conclusion:**

In this study, miR-4443 is found to promote radiotherapy resistance in ESCC cells by regulating PTPRJ expression, which provides a new perspective and clue to alleviate radioresistance.

**Supplementary Information:**

The online version contains supplementary material available at 10.1186/s12967-022-03818-5.

## Introduction

Esophageal cancer, as a global cancer burden, is one of the most aggressive digestive tract malignancies [1]. According to histological type, esophageal cancer is primarily divided into esophageal adenocarcinoma and esophageal squamous cell carcinoma (ESCC), of which 90% are ESCC in China [2]. Because esophageal cancer is insidious in its onset, more than 80% of patients lose the opportunity for surgical treatment at the time of presentation, and concurrent chemoradiotherapy is the standard treatment modality for inoperable esophageal cancer [3]. Despite recent significant advancements in radiotherapy equipment and techniques, the majority of patients still experience local recurrence as a result of radiotherapy resistance, and the 5-year survival rate is less than 30% [4]. Therefore, it is essential to elucidate the specific mechanism of radioresistance, which can provide new perspectives for the radiosensitization therapy of ESCC.

With the development of biological information technology, non-coding RNAs have attracted much attention as a consequence of their emerging role in cancer, of which microRNAs (miRNAs) are a class of non-coding RNAs composed of approximately 22 nucleotides that are responsible for post-transcriptional modification of genes by binding to the 3′UTR of messenger RNA [5]. MiRNAs, novel cancer biomarkers, can play regulatory roles by acting as oncogenes or suppressors [6]. MiRNAs play a pivotal role in radiotherapy, modulating tumor radiosensitivity by taking part in DNA repair, cell cycle analysis, apoptosis, autophagy, and ferroptosis [7]. As an example, Zheng and coworkers revealed that miR-640 potentiated glioma radiosensitivity by focusing on SLIT1 to repress the Wnt/β-catenin flagging pathway [8]. Recent work by McGrath and colleagues found that manipulating miR-31 expression altered the radiosensitivity of pancreatic ductal adenocarcinoma cells by controlling oxidative pressure [9]. In addition, it has been reported that miR-222 focusing on PTEN, and miR-155 focusing on FOXO3a actuated radiation opposition in colorectal malignant growth [10]. MiRNAs have been found to modulate the esophageal cancer response to radiation therapy. Chen et al. found that miR-450a-5p suppressed autophagy and enhanced radiosensitivity in esophageal squamous cell carcinoma by targeting dual-specificity phosphatase 10 [11]. It has been reported that miR-196b promotes chemoradiotherapy resistance of esophageal squamous cell carcinoma by inhibiting EPHA7 [12]. In our previous study, Jin et al. found that upregulation of microRNA-98 increased radiosensitivity in esophageal squamous cell carcinoma [13]. However, it is still unclear how miRNAs function as latent radiosensitizers in ESCC, and further discovery of miRNAs affecting ESCC radiosensitivity is still needed.

MiR-4443, located on chromosome 3p21.31, is a recently recognized miRNA that has been distinguished as a basic controller of tumorigenesis and metastasis [14, 15]. The expression levels and regulatory mechanisms of miR-4443 were not completely consistent in different tumor types. For instance, miR-4443 was upregulated in breast cancer [16] and downregulated in papillary thyroid carcinoma and ovarian cancer [14, 17]. Intriguingly, it has been discovered that exosomes carrying miR-4443 increase cisplatin resistance in non-small cell lung cancer by modulating ferroptosis caused by FSP1 m6A modification [18]. Recent work by Wang et al. revealed that miR-4443 was elevated in ESCC [19]. Nonetheless, there has been little significant understanding of the role of miR-4443 in the ESCC response to radiotherapy, and further elucidation is needed.

Therefore, to explore whether miR-4443 is associated with radiosensitivity and other biological characteristics of ESCC, we established an acquired radioresistant ESCC cell line in which miR-4443 expression was detected. The effect of miR-4443 on radiosensitivity in ESCC was investigated by regulating miR-4443 expression levels up and down by genetic manipulation. In this study, miR-4443 was found to be upregulated in radioresistant ESCC cells. MiR-4443 regulated ESCC radiosensitivity by influencing DNA damage repair, apoptosis, and G2 cycle arrest. Our discoveries reveal that miR-4443 targets PTPRJ to augment radioresistance in ESCC, providing a new basis for developing new therapeutic strategies to improve radiosensitivity in ESCC.

## Methods

### Cell culture

Human ESCC cell lines (Eca-109, KYSE-150, and TE-1) were purchased from the Cell Bank of the Chinese Academy of Sciences Typical Culture Preservation Committee (Shanghai, China). The human normal esophageal cell line (Het-1A) was purchased from ATCC (American Type Culture Collection, Manassas, USA). All ESCC cells were cultured in RPMI 1640 (Gibco, CA, USA) medium with 10% fetal bovine serum (FBS; Gibco, CA, USA) and the Het-1A cell line was cultured in DMEM (Gibco, CA, USA) medium with 10% FBS in a humid atmosphere containing 5% CO_2_ at 37 °C.

### Establishment of radioresistant ESCC cells

The parental KYSE-150 cells were seeded into 6-well plates and cells were initially irradiated with 2 Gy X-ray when the cell density reached 50–60%. After radiation, the culture medium was renewed. When the cells achieved 90% confluence, they were split 1:3 into new 6-well plates and received radiation again. These procedures were repeated 30 times to a total dose of 60 Gy. The surviving cells were characterized as radioresistant cells (termed KYSE-150R cells). The generation of radioresistant cells was validated by performing colony formation assays [20].

### RNA extraction and quantitative real-time polymerase chain reaction (qRT-PCR)

Total RNA was extracted from cells using TRIzol (TaKaRa, Dalian, China) and reverse-transcribed into cDNA with a Prime Script™ RT reagent kit (TaKaRa). MicroRNA-specific cDNAs were generated using a Mir-X™ miRNA First Strand Synthesis kit (TaKaRa). qRT-PCR was carried out using TB Green^®^ Premix Ex Taq™ II (TaKaRa). We normalized the relative miRNA and mRNA expression to U6 and GAPDH, respectively. All experiments were repeated in triplicate. The primer sequences used in this study are listed in Additional file 2: Table S1.

### Cell transfection

Lentivirus particles were designed and purchased from GenePhama (Shanghai, China), including LV2N-hsa-miR-4443 mimics, LV2N-hsa-miR-4443 inhibitor sponge, and controls. PTPRJ small interfering RNA (siRNA) (siPTPRJ), siRNA negative control (siNC), PTPRJ overexpression plasmid (PTPRJ), and empty vector (vector) were purchased from Sangon (Shanghai, China). Transfection was performed according to the manufacturer’s instructions. The target sequences are presented in Additional file 3: Table S2.

### Clonogenic survival assay

Single-cell suspensions were inoculated into six-well plates at densities of 900–12,000 cells per well (TE-1: 0 Gy: 900 cells; 2 Gy: 1500 cells; 4 Gy: 3000 cells; 6 Gy: 9000 cells; and 8 Gy: 12,000 cells; KYSE150/150R: 0 Gy: 900 cells; 2 Gy: 1200 cells; 4 Gy: 1800 cells; 6 Gy: 3000 cells; and 8 Gy: 6000 cells). After cell adherence, they were subjected to 0, 2, 4, 6, or 8 Gy X-ray irradiation, respectively. After 10–14 days of incubation, during which the cell media was replaced every three days, the cells were rinsed with PBS, fixed with methanol, and stained with crystal violet. Colonies containing more than 50 cells were counted and analysed based on the corresponding numbers of initial inoculated cells. Cell survival curves based on the mean survival fractions of the cell line were fitted to a Single-hit multitarget model: SF = 1 − (1−e^(−kD)^)^N^.

### Cell counting kit-8 (CCK-8) assay

In the exponential growth phase, two thousand cells per well were seeded into 96-well plates (100 μl/well). After adhering to the cells, the cells were treated with a single dose of 6 Gy (day 0). At the specified time point (day 0, day 1, day 2, day 3, day 4, day 5), 10 μl CCK-8 solution (UElandy, Suzhou, Jiangsu, China) was added to each well and then incubated for 1 h. The optical density (OD, 450 nm) values were measured by a microplate reader.

### Western blot

Total protein was separated from cell lysates on ice using a radioimmunoprecipitation assay (RIPA) containing protease and phosphatase inhibitors (HEART, Xi’an, Shaanxi, China). We quantified protein levels using an Enhanced BCA Protein Assay Kit (Beyotime, Shanghai, China). Equal amounts of protein were separated on 6 or 12% SDS-PAGE gels (Beyotime) and transferred to PVDF membranes (Millipore, Billerica, MA, USA), which were incubated with primary antibodies at 4 °C overnight. The primary antibodies used in this study were: γH2AX (1:1000, #80312, Cell Signaling Technology, Danvers, MA, USA), PTPRJ (1:5000, ab181244, Abcam, Cambridge, MA), and β-tubulin (1:10,000, AP0064, Bioworld, USA,). After washing four times with Tris-Buffered Saline and Tween 20 (TBST) buffer, the membranes were incubated with horseradish peroxidase (HRP)-conjugated goat anti-rabbit (1:2500, bs-0295G-HRP, Bioss, Beijing, China) or goat anti-mouse immunoglobulin (Ig)G (1:2500, bs-0296G-HRP, Bioss, Beijing, China) for 1 h at room temperature was performed. The protein bands were visualized using chemiluminescence kits (Millipore, Billerica, MA, USA).

### Immunofluorescence

The cells were seeded on 20 × 20 mm glass-bottom round dishes (Corning, NY, USA) and irradiated with a single dose of 8 Gy after adherence. Then, 24 h after irradiation, the cells were washed with phosphate-buffered saline (PBS) and fixed with 4% paraformaldehyde for 20 min at room temperature. Cells were subsequently permeabilized with 0.1% Triton X-100 before being blocked with 5% bovine serum albumin (BSA) for 30 min at room temperature. The cells were stained overnight with Phospho-Histone H2AX (Ser139) (D7T2V) mouse monoclonal antibody at 4 °C, washed three times in PBS, and further incubated with fitc-coupled second antibody (1:300, Proteintech, China) at room temperature for 1 h. Antifade mounting medium with DAPI (Beyotime Biotechnology, Shanghai, China) was utilized to stain nuclei for 10 min, and then a Leica TC5 SP5 confocal microscope was adopted to visualize and image the cells.

### Apoptosis and cell cycle analyses

Cells were first seeded into a 6-well plate at a density of 1 × 10^5^ cells per well and treated with 6-MV X-ray radiation at doses of 0 Gy and 8 Gy. Cells were collected and stained with an Annexin V-APC/7-AAD double staining apoptosis detection kit (LiankeBio, Hangzhou, China) according to the manufacturer's instructions. For cell cycle assays, cells were harvested and stained with PI solution (KeyGen Biotech, Nanjing, China) with RNase A. Flow cytometry was used to evaluate the luciferase intensity at 24 h after treatment.

### Bioinformatic analysis

The GSE43732 miRNA microarray dataset and the GSE53624 gene expression microarray dataset of 119 ESCC patients were downloaded from the Gene Expression Omnibus database (http://www.ncbi.nlm.nih.gov/geo/) (Additional file 4: Table S3). The maximum expression was considered to be the expression of genes with several probes. The ESCC patients’ transcription profile and clinical data of The Cancer Genome Atlas (TCGA) (http://cancergenome.nih.gov/) cohort were analyzed as previously described [21]. Weighted gene co-expression network analysis (WGCNA) based on the FPKM (fragments per kilobase of transcript per million mapped reads) values of TCGA-ESCC data was used to explore the relationships between gene networks and diseases as well as correlations between gene modules and clinical characteristics.

### Statistical analysis

GraphPad Prism 8.0 and R 4.2.1 software were used for statistical analyses and data visualization. The data are expressed as the mean ± standard deviation (SD). P < 0.05 indicated statistical significance. Differences between groups were analysed using Student’s t-test, Wilcoxon test, or one-way ANOVA.

## Results

### MiR-4443 is upregulated in radioresistant ESCC cells

**Fig. 1:**
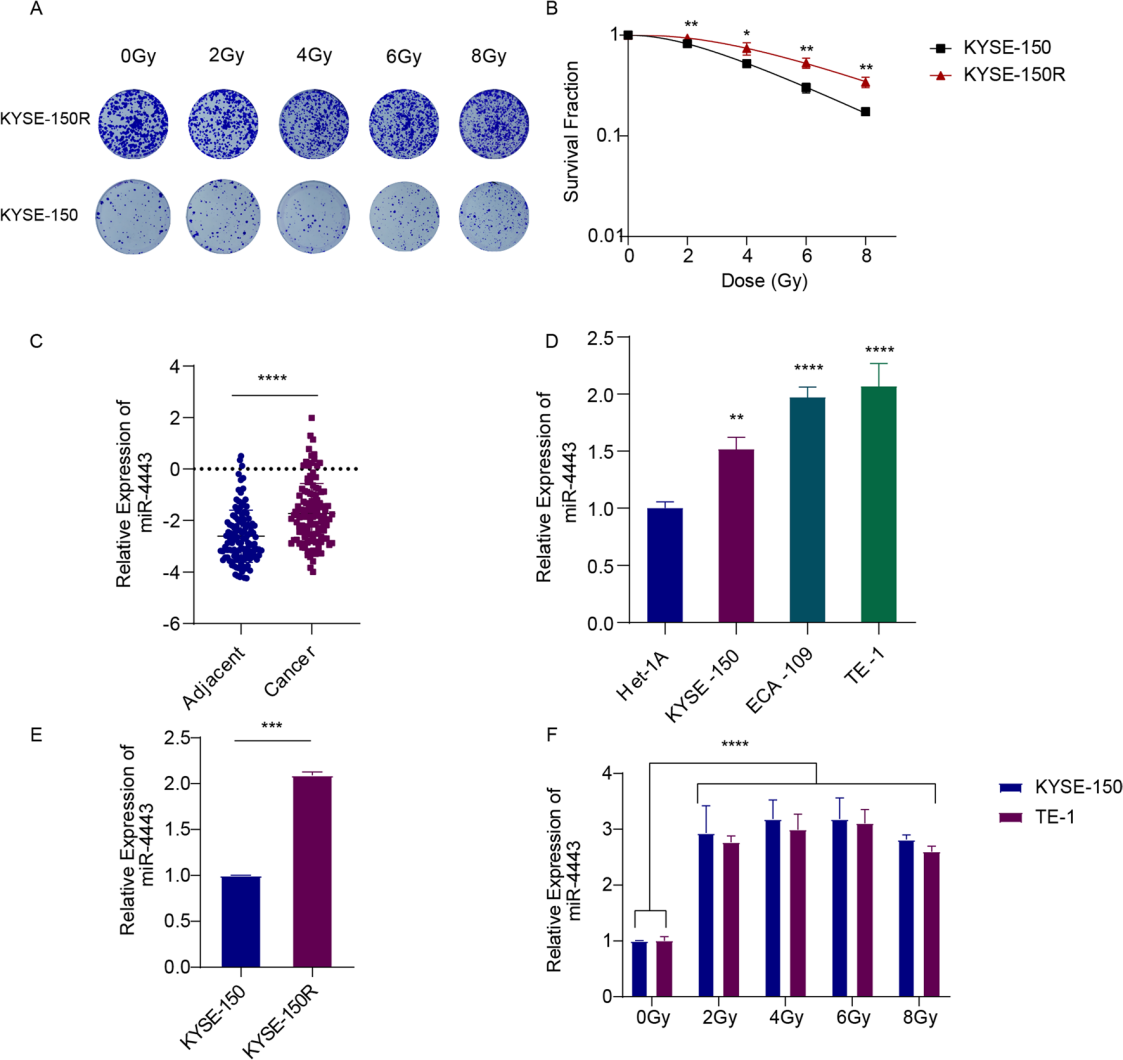
MiR-4443 was upregulated in radioresistant ESCC cells. **A** Clonogenic survival assays were conducted using KYSE-150R and parental KYSE-150 cells to evaluate the radioresistance of KYSE-150R. **B** Survival curves of KYSE-150R cells and KYSE-150 cells were calculated and fitted to a multitarget model. **C** The expression profiles of miR-4443 in GSE43732 (Adjacent, n = 119; Cancer, n = 119). **D** qRT-PCR for miR-4443 in 3 esophageal squamous cell carcinoma cell lines and 1 normal esophageal cell line. **E** qRT-PCR for miR-4443 in parental cells and radioresistant cells. **F** qRT-PCR analysis of miR-4443 expression in ESCC cells treated with different doses of radiation. Data are shown as the mean ± SD from three independent experiments. (*p < 0.05, **p < 0.01, ***p < 0.001, ****p < 0.0001)

To explore the underlying mechanism of radiation resistance in esophageal squamous cell carcinoma, we established an acquired radioresistant ESCC cell line (named KYSE-150R) using the protocol described in the methods section. The results of the cell colony formation assay and survival fraction of parental KYSE-150 cells and radioresistant KYSE-150R cells are shown in Fig. 1A, B. The results showed that the survival fraction of KYSE-150R was significantly higher than that of KYSE-150, which indicated that KYSE-150R had higher radioresistance characteristics than KYSE-150. We extracted the expression profile data of miR-4443 in GSE43732 and found that miR-4443 was highly expressed in tumor tissues compared with adjacent tumor tissues (Fig. 1C). The qRT-PCR results showed that the expression of miR-4443 was higher in ESCC cell lines than in normal esophageal cell line (Het-1A) (Fig. 1D). The expression of miR-4443 was upregulated in KYSE-150R cells compared to KYSE-150 cells (Fig. 1E). After irradiation with different doses, miR-4443 expression was approximately two–threefold higher in KYSE-150 and TE-1 cells, respectively (Fig. 1F).

### MiR-4443 regulates the radioresistance of ESCC cells

**Fig. 2:**
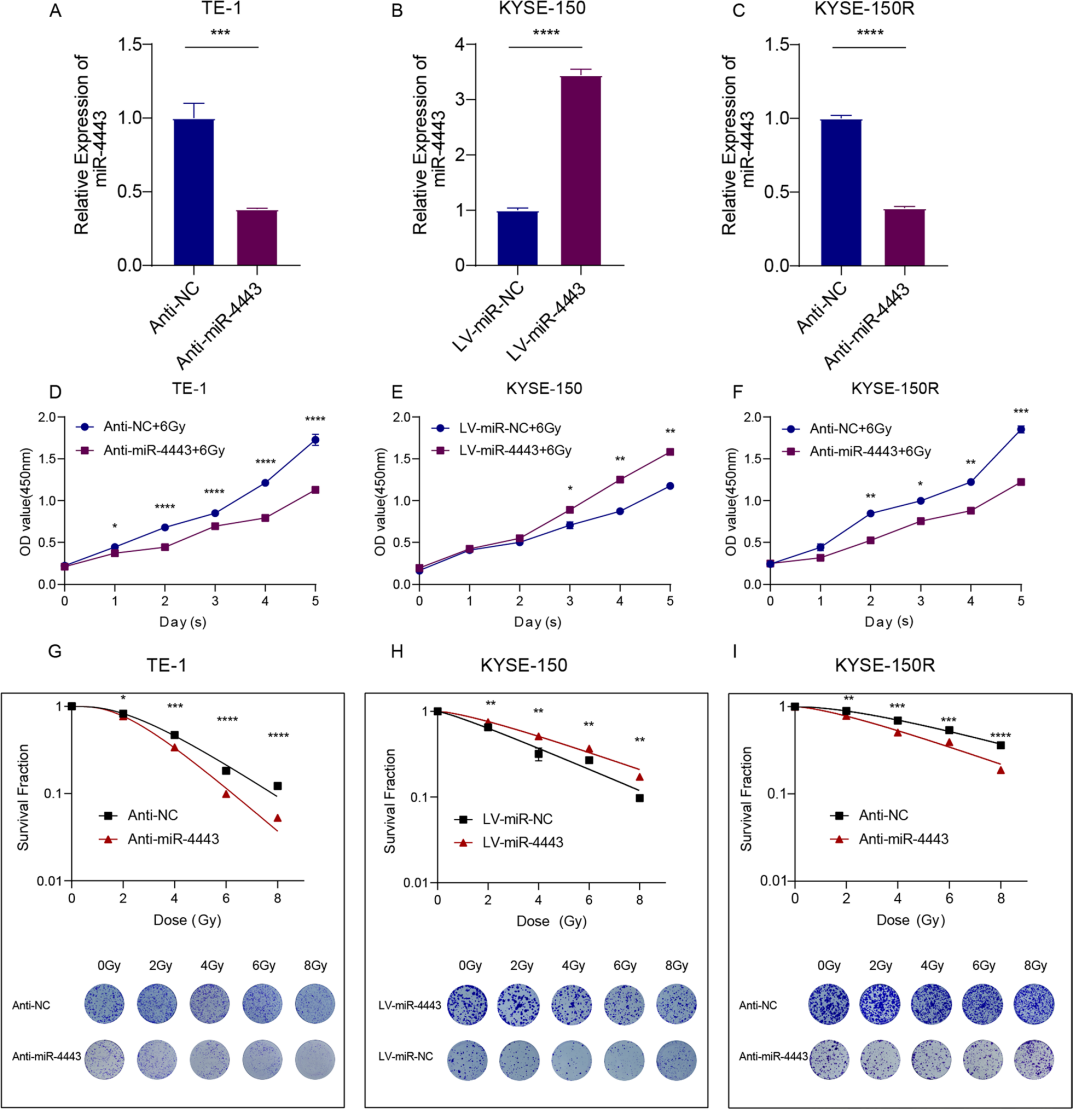
MiR-4443 promoted the proliferation of ESCC cells after irradiation. **A**–**C** Transfection efficiency was validated by qRT-PCR. **D**–**F** CCK-8 assays were performed in indicator cells to determine the cell proliferation rates after irradiation. **G**–**I** Clonogenic survival assays and the corresponding survival fraction curves of indicator cells following exposure to 0, 2, 4, 6 and 8 Gy of X-ray. Data are presented as the mean ± SD; n = 3 independent experiments. (*p < 0.05, **p < 0.01, ***p < 0.001, ****p < 0.0001)

To investigate the role of miR-4443 in ESCC radiation resistance, we decreased the expression of miR-4443 in TE-1 and KYSE-150R cells by transfection of anti-miR-4443 lentivirus and upregulated the expression of miR-4443 in KYSE-150 cells by transfection of miR-4443 mimics lentivirus. Transfection efficiency was determined by qRT-PCR, and the results showed that upregulation and downregulation of miR-4443 expression were appropriate (Fig. 2A–C). The CCK-8 method was used to detect the role of miR-4443 expression in cell growth after irradiation. Compared with the NC group, TE-1 cells and KYSE-150R cells with miR-4443 inhibition showed significant growth inhibition after irradiation, while the overexpression of miR-4443 in KYSE-150 cells had the opposite effect (Fig. 2D–F). We next conducted clonogenic survival assays to further study the role of miR-4443 in the radiation resistance of ESCC cells. We found that miR-4443 inhibition significantly inhibited the survival fraction of TE-1 and KYSE-150R cells under different doses of irradiation, while the overexpression of miR-4443 markedly enhanced the survival fraction of KYSE-150 cells subjected to different doses of irradiation (Fig. 2G–I).

**Fig. 3:**
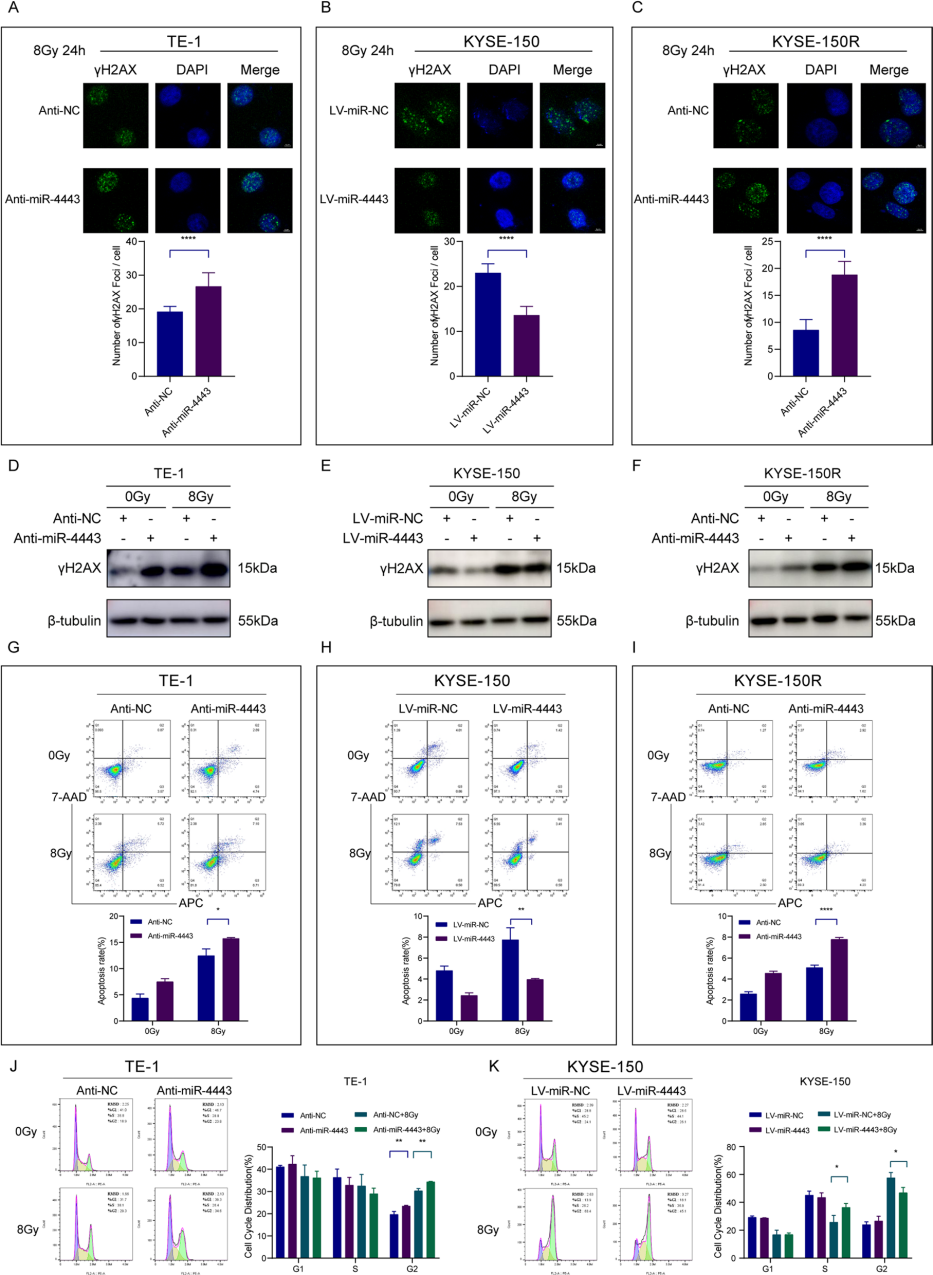
Knockdown of miR-4443 enhanced the radiosensitivity of ESCC cells. **A**–**C** Representative immunofluorescence images of nuclear γ-H2AX foci (cell nuclei: blue; γ-H2AX foci: green) in the indicated cells after 24 h of 8 Gy radiation. Scale bar: 4 µm. **D**–**F** Western blot analysis of γ-H2AX protein expression levels in the indicated cells after 24 h of 8 Gy radiation. **G**–**I** Evaluation of the apoptosis rates of ESCC cells with altered expression levels of miR-4443. **J**, **K** Cell cycle distribution in the indicated cells after 6 h of 8 Gy radiation. Data are presented as the mean ± SD; n = 3 independent experiments. (*p < 0.05, **p < 0.01, ***p < 0.001, ****p < 0.0001)

The detection of γH2AX has become the most commonly used method for quantifying double-strand breaks and their repair kinetics. The γH2AX foci accumulated rapidly after IR and peaked at 30 min. At 24 h, the residual lesions showed persistent DNA damage, indicating increased radiosensitivity [22]. The number of γ-H2AX foci in TE-1, KYSE-150, and KYSE-150R cells after irradiation was detected by immunofluorescence. The results showed that the number of γH2AX foci increased in miR-4443 silenced cells, but decreased in miR-4443 overexpressing cells at 24 h after irradiation (Fig. 3A–C). In addition, γ-H2AX protein expression was increased in TE-1 or KYSE-150R cells with miR-4443 inhibition after irradiation. However, the upregulation of miR-4443 in KYSE-150 cells decreased the expression of γ-H2AX protein (Fig. 3D–F, Additional file 1: Fig. S1A-C). The miR-4443-knockdown TE-1 or KYSE-150R cells had a greater rate of apoptosis than the controls when subjected to radiation stress (8 Gy). The miR-4443-over-expressing cells, however, displayed the opposite outcomes (Fig. 3G–I). Additionally, cell cycle distribution analysis revealed that the combined effect of irradiation and miR-4443 knockdown significantly induced G2/M arrest in esophageal cancer cells, whereas miR-4443 overexpression reduced G2/M block in cells after irradiation (Fig. 3J, K). These results indicated that inhibition of miR-4443 could increase the level of DNA damage and sensitization of ESCC cells to irradiation.

### Key gene modules relevant to radiosensitivity were identified by WGCNA

**Fig. 4:**
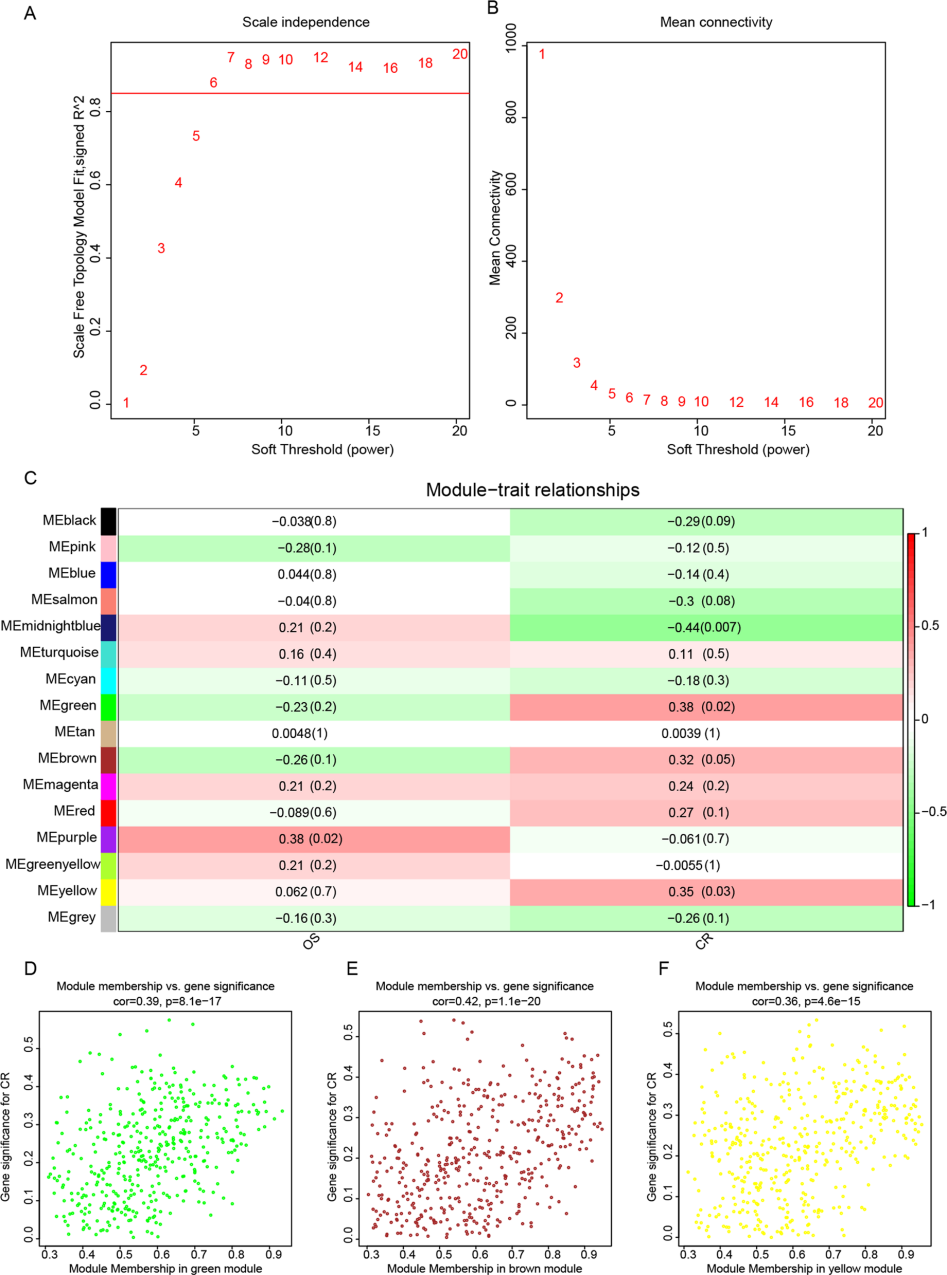
Identification of key modules correlated with radiosensitivity in TCGA-ESCC through WGCNA. **A** Analysis of the scale-free fit index for various soft-thresholding powers (β = 6). **B** Analysis of the mean connectivity for various soft-thresholding powers. **C** Heatmap of the associations between module eigengenes and clinical characteristics based on TCGA-ESCC. **D**–**F** Scatter plots of module eigengenes in the green, brown, and yellow modules. The figures were created using R software v4.2.1. OS (overall survival), CR (complete response)

We used the WGCNA method to identify genes associated with radiosensitivity. Thirty-six patients with ESCC who received radiotherapy and had radiotherapy response evaluation data were downloaded from the TCGA database. According to the radiotherapy response, 24 patients were divided into the radiosensitive group (complete response, CR) and 12 patients were divided into the radioresistant group (partial response, stable disease or progressive disease). The clinical characteristics of the two groups are shown in Additional file 1: Fig. S2A and Additional file 5: Table S4. After quality assessment, a scale-free network was constructed by selecting β = 6 (scale-free R^2^ = 0.8) (Fig. 4A, B). Next, similar modules were merged to trim genes whose correlation with the module eigengene was less than the defined threshold (min Module Size of 30 and merge Cut Height of 0.25) (Additional file 1: Fig. S2B). Fifteen modules were identified in addition to the gray module, among which the green module (r = 0.38, p = 0.02, n = 423), brown module (r = 0.32, p = 0.05, n = 451), and yellow module (r = 0.35, p = 0.03, n = 445) were significantly positively correlated with the CR phenotype (Fig. 4C). Figure 4D–F shows the correlation between module membership and gene significance of complete response and their respective p-values for the green module, brown module, and yellow module. Therefore, genes positively correlated with the CR phenotype were selected from these three modules for subsequent analysis (Additional file 6: Table S5).

### PTPRJ is identified as the target gene of miR-4443

**Fig. 5:**
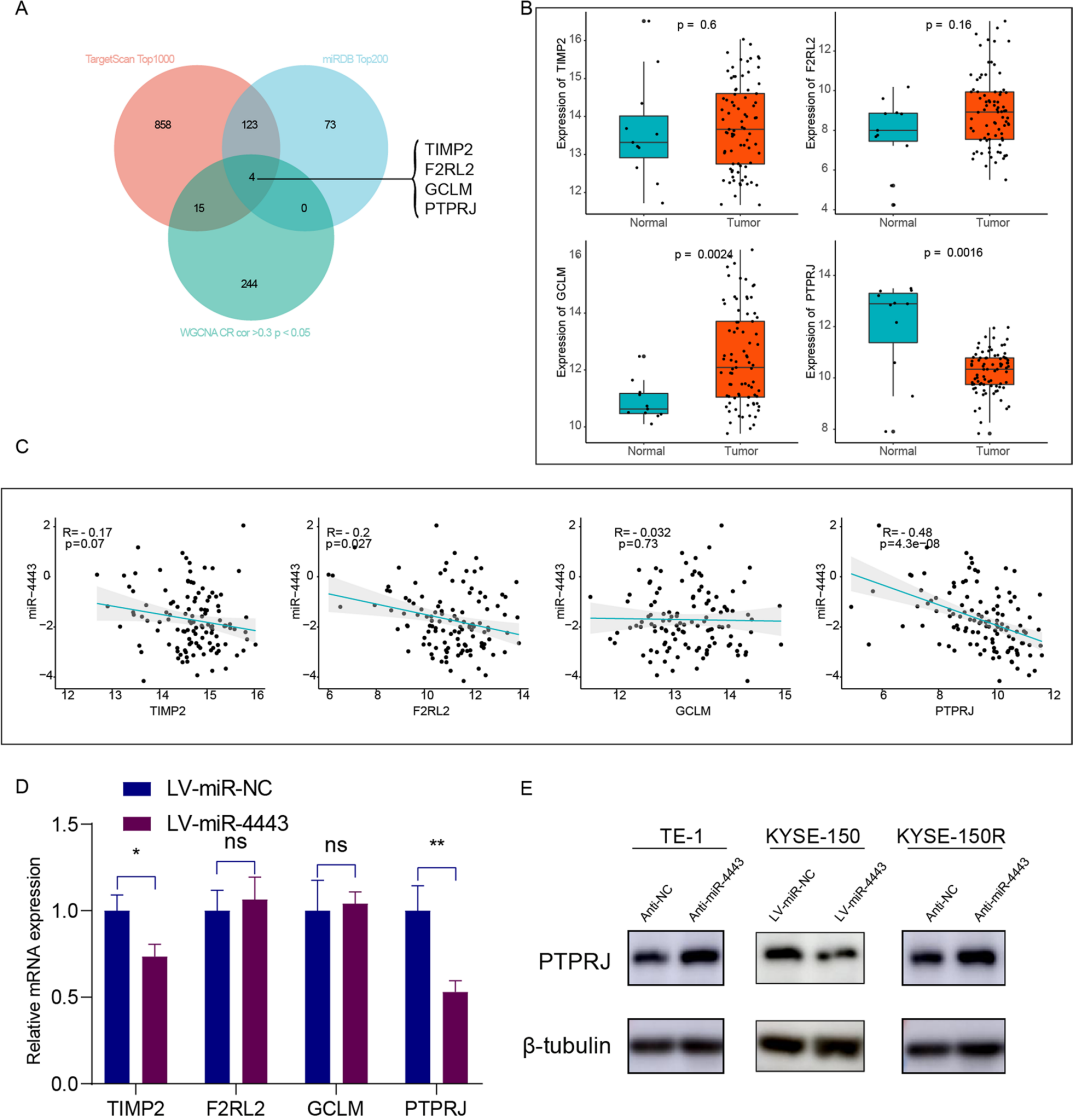
PTPRJ was a potential target gene of miR-4443. **A** The Venn diagram showed the number of miR-4443 target genes predicted by the two miRNA databases (TargetScan and miRDB) and associated with radiosensitivity. **B** The expression patterns of potential target genes of miR-4443 in the TCGA-ESCC cohort [ESCC tissue (n = 81) vs normal tissues (n = 11)]. **C** The correlation between the expression levels of miR-4443 and its potential targets in 119 ESCC specimens. The correlation test was conducted by the Spearman coefficient. **D** qRT-PCR for potential target genes of miR-4443 in the indicated cells. **E** The protein levels of PTPRJ in the indicated ESCC cells. Mean ± SD, N = 3, (*p < 0.05, **p < 0.01)

MiRNAs block translation or lead to mRNA degradation by binding partial or complete sequence homology to the 3'UTR of the target mRNA [6, 23]. To investigate the potential molecular mechanisms by which miR-4443 regulates radioresistance in ESCC cells, we used TargetScan and the miRDB database to predict the putative targets of miR-4443 (Additional file 7: Table S6). Subsequently, we selected the top 1000 predicted genes in TargetScan and the top 200 predicted genes in miRDB to intersect with the radiation-sensitive genes in WGCNA, and a total of 4 candidate genes were screened (Fig. 5A). We analysed the expression of these four genes in the TCGA-ESCC dataset and found that only PTPRJ was expressed at low levels in the tumor tissue (Fig. 5B). Subsequently, we extracted the expression profile data of these four genes from the GSE53624 microarray dataset and correlated them with the expression data of miR-4443, and the results showed that the expression of PTPRJ was negatively correlated with the expression of miR-4443 (Fig. 5C). qRT-PCR and western blot assays showed that the mRNA and protein expression levels of PTPRJ in the miR-4443-over-expressing cells were significantly lower than those in the control cells (Fig. 5D, E). Furthermore, PTPRJ protein levels increased in miR-4443-knockdown cells. These findings suggested that PTPRJ was a target gene of miR-4443 in ESCC cells.

### PTPRJ is required for the miR-4443-mediated ESCC cell response to irradiation

**Fig. 6:**
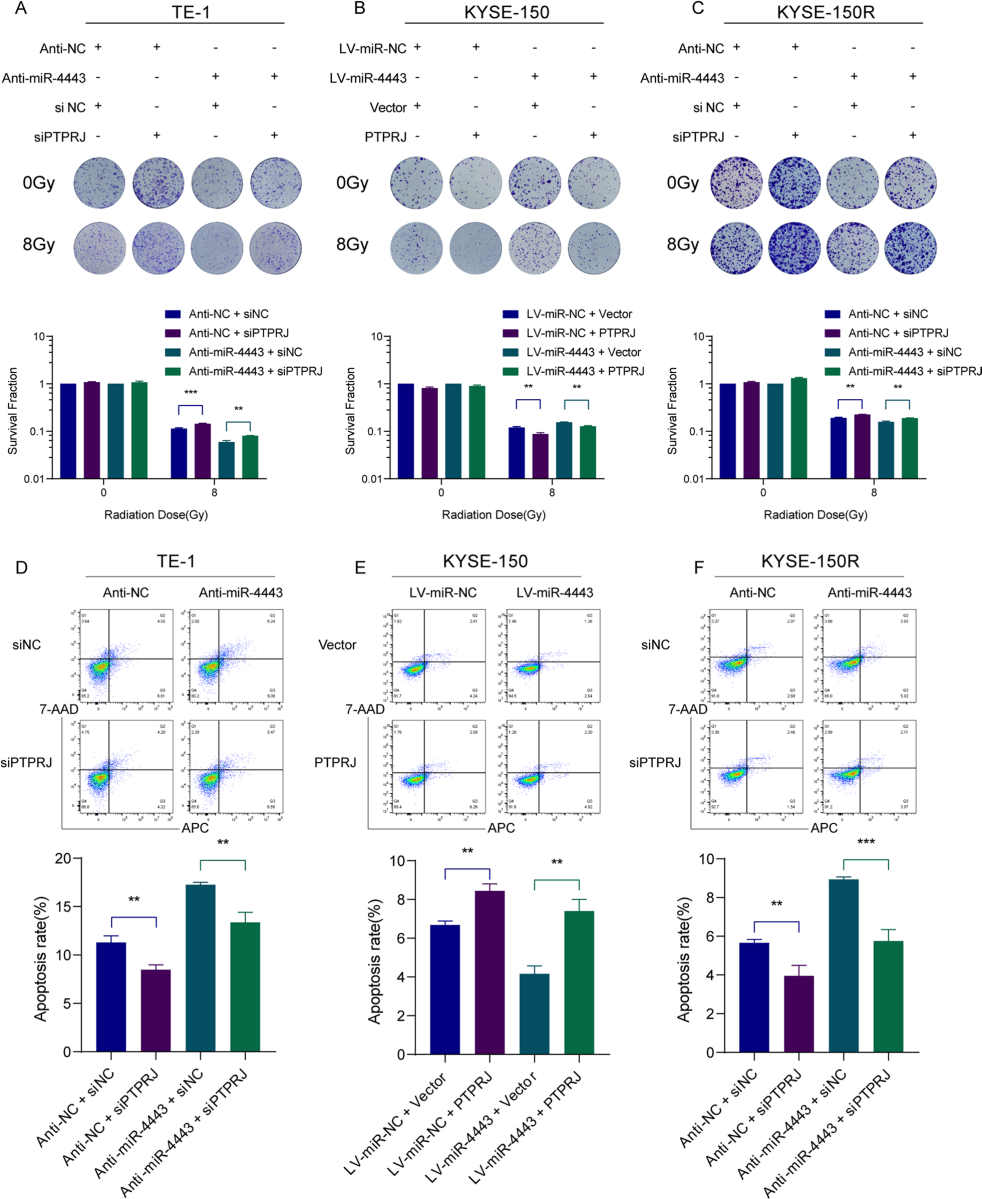
PTPRJ was required for the miR-4443-mediated ESCC cell response to irradiation. **A**–**C** Clonogenic survival assays of the radioresponse in the indicated cells after restoration of PTPRJ and depletion of PTPRJ. **D**–**F** After 8 Gy irradiation, the apoptosis rates were evaluated in the indicated cells after restoration of PTPRJ and depletion of PTPRJ. Data are presented as the mean ± SD; n = 3 independent experiments. (*p < 0.05, **p < 0.01, ***p < 0.001)

We carried out rescue tests to determine whether PTPRJ could reverse the miR-4443-induced ESCC cell response to radiation. First, we confirmed that PTPRJ overexpression reversed the effect of miR-4443 upregulation on ESCC cell survival following radiation while inhibition of PTPRJ had the opposite effect (Fig. 6A–C). Subsequently, inhibition of PTPRJ in TE-1 and KYSE-150R cells significantly restored the increased apoptosis induced by miR-4443 knockdown after irradiation (Fig. 6D, F). In addition, the upregulation of PTPRJ in KYSE-150 cells reversed the effect of miR-4443 overexpression (Fig. 6E). These results indicated that miR-4443 reduced the apoptosis level of ESCC cells after radiation and enhanced the resistance of ESCC cells to radiation by directly inhibiting the expression of PTPRJ.

## Discussion

One of the deadliest cancers in the world is esophageal cancer, and ESCC is the most predominant pathological subgroup. The key factor limiting the effectiveness of radiotherapy for ESCC is radiotherapy resistance. The development of radioresistance is a multigene, multifactor, and multimechanism process. Various factors such as DNA damage repair, cell cycle arrest, tumor microenvironment (including hypoxia and angiogenesis), regulation of autophagy and the presence of tumor stem cells, have been discovered to contribute to tumor radioresistance [24–26]. Notwithstanding, the molecular mechanism of radioresistance in ESCC remains unclear. It is of great scientific value and clinical significance to deeply explore the role of radioresistance and its molecular mechanism to develop radiosensitization strategies targeting key molecules in ESCC.

It has recently been discovered that particular miRNAs are differentially expressed in tumor tissue and normal tissue, which has implications for tumor growth and radiation resistance. MiR-4443 has been identified as a critical regulator of tumorigenesis. By way of illustration, miR-4443 was highly expressed in epirubicin-resistant cell lines, inhibited their apoptosis, and induced malignant progression in breast cancer and non-small cell lung cancer [16, 27]. However, miR-4443 is also considered to be a tumor suppressor, inhibiting tumor proliferation, migration and invasion, such as in papillary thyroid cancer, glioblastoma and colorectal cancer [15, 17, 28]. MiR-4443 has already been reported to be highly expressed in ESCC, but whether it affects radiosensitivity in ESCC is unclear. In this study, we established an acquired radioresistant ESCC cell line (KYSE-150R) with a total radiation dose of 60 Gy in reference to the clinical radiotherapy guideline for esophageal squamous cell carcinoma. We demonstrated for the first time that miR-4443 was highly expressed in radioresistant ESCC cells compared to parental cells and that radiation can induce miR-4443 expression. Functional experiments showed that overexpression of miR-4443 significantly enhanced the survival fraction of KYSE-150 cells subjected to different doses of irradiation, while downregulation of miR-4443 inhibited the survival fraction of TE-1 and KYSE-150R cells under different doses of irradiation. This suggested that miR-4443 was a potential therapeutic target for radioresistant ESCC.

It is well-known that DNA is a target of radiation damage and that radiation can damage DNA by acting directly or indirectly [7]. In DNA damage, cells initiate complex biochemical signal transduction pathways that recognize the type of damage, initiate corresponding repair pathways, repair the damage, or lead to cellular senescence, autophagy, apoptosis, and if not effectively repaired, cell death [29]. Radiosensitivity is associated with DNA damage repair and miRNAs. It has been reported that miR-146a-5p inhibits the expression of binding replication protein A3 to activate the DNA damage repair pathway, thereby promoting radiosensitivity in hepatocellular carcinoma [30]. Serine 139 on H2AX is phosphorylated and modified to form phosphorylated H2AX, namely, γH2AX, which is a marker of DNA double-strand breaks and can reflect the degree of DNA damage and repair.​ Our results demonstrated that the upregulation of miR-4443 decreased γH2AX expression in ESCC cells at 24 h after irradiation, whereas the downregulation of miR-4443 increased γH2AX expression. Another promising finding was that silencing miR-4443 resulted in a greater apoptotic rate in TE-1 or KYSE-150R cells after irradiation than in control cells, indicating that inhibition of miR-4443 could increase apoptosis in irradiated ESCC cells. After the cells were damaged by ionizing radiation, the cells were arrested at various stages of the cell cycle, so that the cells had enough time to repair themselves and evade radiation damage, thus showing radiation resistance. The cell cycle phase affects cell radiosensitivity. Cells in the G2/M phase are the most sensitive to radiation, and the sensitivity decreases as the cell cycle progress from the G1 to the S phase. Our study found that miR-4443 downregulation promoted G2/M arrest in irradiated ESCC cells. Based on the above evidence, we demonstrated for the first time that miR-4443 promoted ESCC radioresistance by enhancing DNA damage repair, inhibiting apoptosis and decreasing the proportion of cells in the G2/M phase.

Direct and negative regulation of downstream targeted mRNAs is one way in which miRNAs exert their biological function. In this study, WGCNA was used to identify genes associated with radiosensitivity and then intersected with the prediction results of the TargetScan and miRDB databases to predict the putative targets of miR-4443. In addition, by bioinformatics methods, we found that PTPRJ expression was low in tumor tissues and that its expression was negatively correlated with miR-4443 expression. The wet experiment further verified that the mRNA and protein expression levels of PTPRJ in miR-4443-overexpressing cells were significantly lower than those in control cells. PTPRJ was therefore identified as a target gene of miR-4443. PTPRJ is located on chromosome 11p11.2 and encodes a receptor-like protein tyrosine phosphatase, whose aberrant expression plays an important role in disrupting the malignant phenotype of tumor cells [31]. MiRNAs have been reported to regulate the gene expression of PTPRJ and thus affect tumor progression. Zhang et al. found that miR-155 directly bound to the 3′UTR of PTPRJ mRNA and inhibited its expression to regulate the proliferation of colorectal cancer cells [32]. Luo et al. revealed that PTPRJ was significantly downregulated in hepatocellular carcinoma tissues, and miR-328 significantly promoted the migration and invasion of hepatocellular carcinoma cells by suppressing PTPRJ expression [33]. Significantly, Shefler and coworkers revealed that the target gene of miR-4443 was PTPRJ, which was fully consistent with our results [34]. Moreover, the relationship between miR-4443, PTPRJ, and tumor radiotherapy has not been reported. Our findings discovered that PTPRJ reversed miR-4443-induced ESCC cell response to radiation by using rescue studies. In summary, our research demonstrated that miR-4443 reduced the level of apoptosis of ESCC cells after radiation and enhanced the resistance of ESCC cells to radiation by directly inhibiting the expression of PTPRJ.

Nonetheless, this study still has certain restrictions. First, only one type of radioresistant ESCC cell line was established and investigated in this study. Second, these studies were only carried out in vitro, and further verification from animal experiments is lacking. Nevertheless, we preliminarily identified a novel mechanism of miR-4443-mediated tumor radioresistance, offering fresh perspectives for radiosensitization in ESCC. In the future, we will investigate improved techniques to confirm the role of miR-4443 in more radioresistant cell lines and animal trials, and to provide stronger evidence to support the preliminary conclusions of this study.

## Conclusions

Overall, our study demonstrated that miR-4443 promoted radiotherapy resistance in ESCC cells by modulating PTPRJ expression, thus providing a new perspective and clue to alleviate radioresistance.

## Supplementary Information

**Additional file 1: Figure S1.** Knockdown of miR-4443 enhanced the radiosensitivity of ESCC cells. (A) Bar charts of γ-H2AX protein expression levels in the indicated cells after 24 h of 8 Gy radiation. **Figure S2.** WGCNA of TCGA-ESCC dataset. (A) Clustering dendrogram of samples in the TCGA-ESCC dataset. The clustering was based on the RNA-seq data. Colour intensity varies with OS (overall survival) and CR (complete response). In terms of OS, the color changes from white to red, indicating an increase in OS. In terms of CR, red indicates the radiosensitive group, and white indicates the radioresistant group. (B) Dendrogram of all genes clustered based on a dissimilarity measure (1-TOM) in TCGA-ESCC through WGCNA.

**Additional file 2: Table S1.** The sequences of primers used in qRT-PCR.

**Additional file 3: Table S2.** The sequences of siRNA and lentivirus particle used in this study.

**Additional file 4: Table S3.** The list of clinical information of 119 ESCC patients.

**Additional file 5: Table S4.** The list of thirty-six patients with ESCC received radiotherapy.

**Additional file 6: Table S5.** The result of WGCNA.

**Additional file 7: Table S6.** miR-4443 target genes were predicted by TargetScan.

## Data Availability

All data generated was shown in this manuscript. TCGA-ESCC (https://www.cancer.gov/tcga) and the data sets of GSE53624 and GSE43732 from GEO (https://www.ncbi.nlm.nih.gov/geo/) are publicly open and available.
