# Supplementary material for: miR-4443 promotes radiation resistance of esophageal squamous cell carcinoma via targeting PTPRJ
Source: J Transl Med. 2022 Dec 28;20:626. doi: 10.1186/s12967-022-03818-5 (PMC9795664; doi:10.1186/s12967-022-03818-5)
Supplement: Supplementary file 1 — Additional file 1: Figure S1. Knockdown of miR-4443 enhanced the radiosensitivity of ESCC cells. (A) Bar charts of γ-H2AX protein expression levels in the indicated cells after 24 h of 8 Gy radiation. Figure S2. WGCNA of TCGA-ESCC dataset. (A) Clustering dendrogram of samples in the TCGA-ESCC dataset. The clustering was based on the RNA-seq data. Colour intensity varies with OS (overall survival) and CR (complete response). In terms of OS, the color changes from white to red, indicating an increase in OS. In terms of CR, red indicates the radiosensitive group, and white indicates the radioresistant group. (B) Dendrogram of all genes clustered based on a dissimilarity measure (1-TOM) in TCGA-ESCC through WGCNA. [file 12967_2022_3818_MOESM1_ESM.docx]

Additional file 1

**
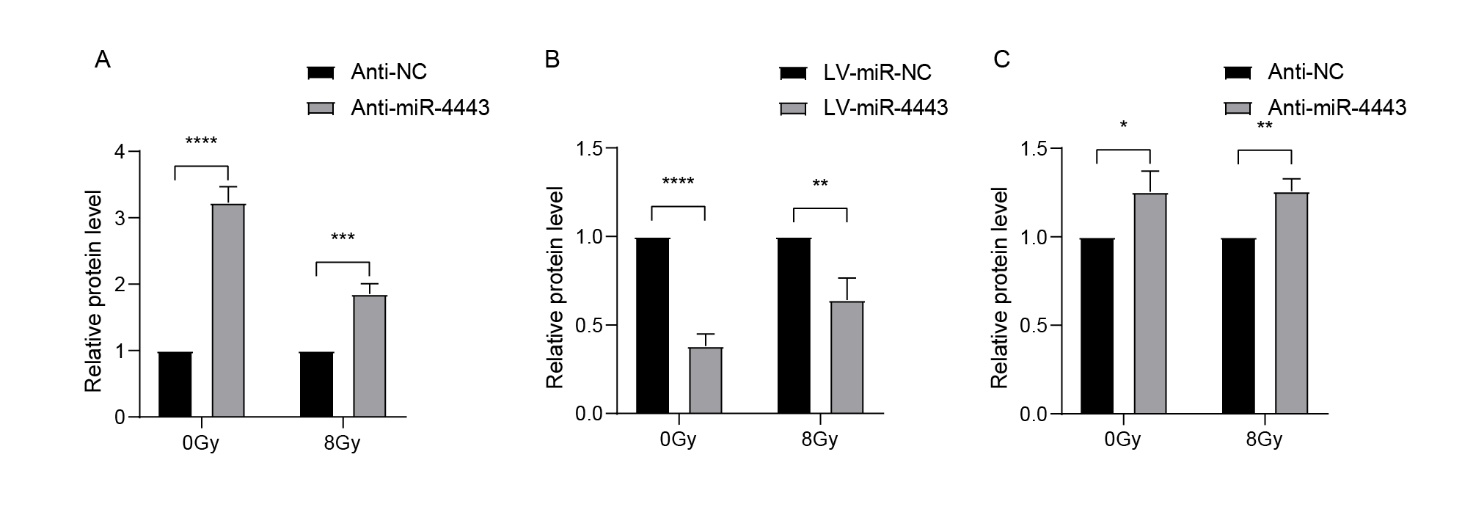
**

**Figure S1.** Knockdown of miR-4443 enhances the radiosensitivity of ESCC cells. (A) Bar charts of γ-H2AX protein expression levels in the indicated cells after 24 h of 8 Gy radiation.

**
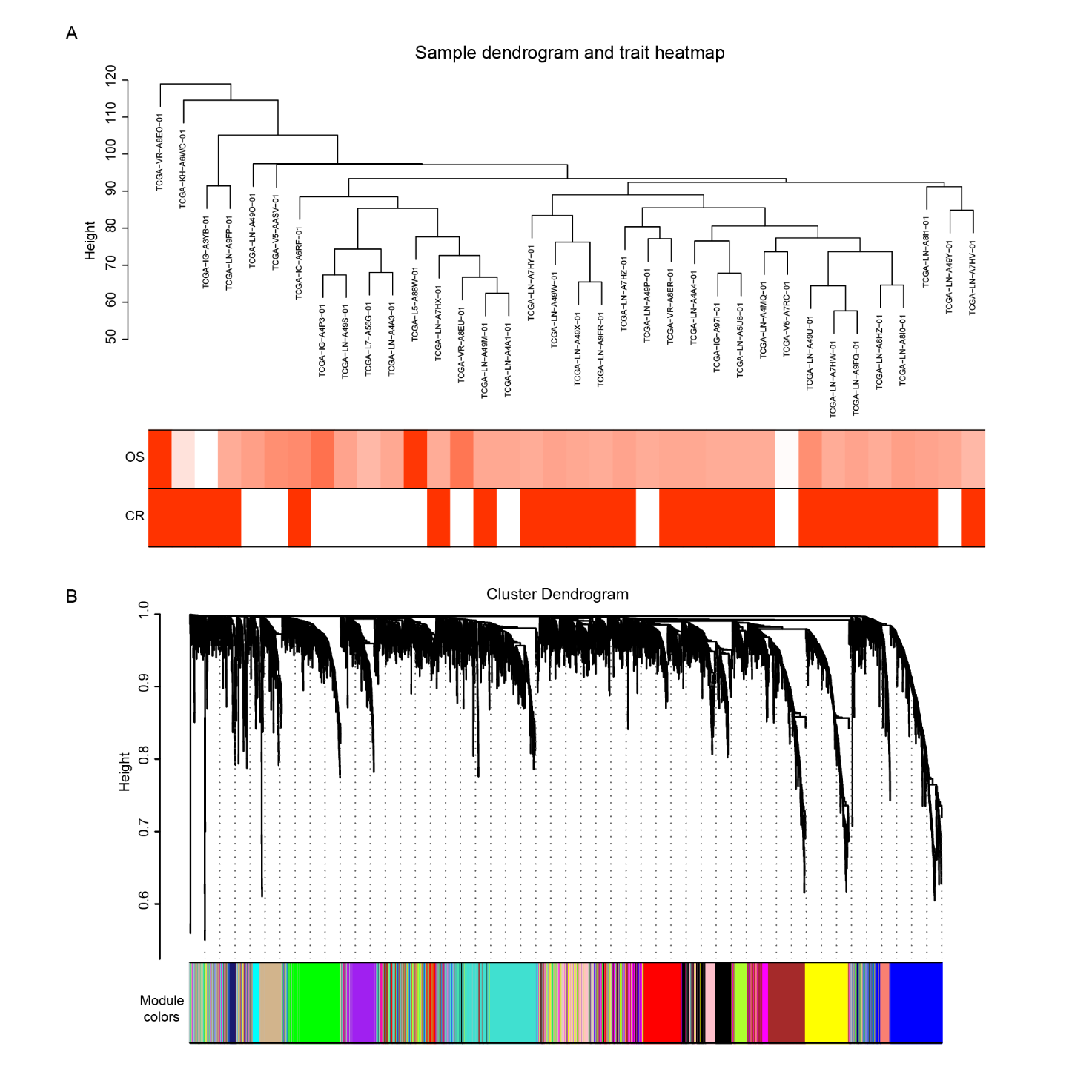
**

**Figure S2.** WGCNA of TCGA-ESCC dataset. (A) Clustering dendrogram of samples in TCGA-ESCC dataset. The clustering was based on the RNA-seq data. Color intensity varies with OS (overall survival) and CR (complete response). In terms of OS, the color changes from white to red, indicating an increase of OS. In terms of CR, red means radiosensitive group, and white indicates radioresistant group. (B) Dendrogram of all genes clustered based on a dissimilarity measure (1-TOM) in TCGA-ESCC through WGCNA.
